# Osteomyelitis and aortic arteritis with thrombosis as primary manifestations of severe paracoccidioidomycosis: a case report

**DOI:** 10.1590/S1678-9946202567044

**Published:** 2025-07-07

**Authors:** Amaro Nunes Duarte-Neto, Katia Cristina Dantas, Suzete Cleusa F. Spina Lombardi, Roseli Santos de Freitas-Xavier, Adriana P. Vicentini, Alfredo Mendroni, Simon Claros Claros, Luiz Fernando Ferraz da Silva, Paulo Hilário Nascimento Saldiva, Marisa Dolhnikoff, Marielton dos Passos Cunha, Thais Mauad

**Affiliations:** 1 Universidade de São Paulo Faculdade de Medicina, Departamento de Patologia, São Paulo, São Paulo, Brazil; 2 Fundação Pró-Sangue, Divisão de Pesquisa - Segurança Transfusional, São Paulo, São Paulo Brazil; 3 Universidade de São Paulo, Faculdade de Medicina, Instituto de Medicina Tropical de São Paulo, São Paulo, São Paulo, Brazil; 4 Instituto Adolfo Lutz, São Paulo, São Paulo, Brazil; 5 Universidade Estadual de Campinas, Instituto de Biologia, Departamento de Genética, Evolução, Microbiologia e Imunologia, Campinas, São Paulo, Brazil

**Keywords:** Osteomyelitis, Aortic arteritis, Thrombosis, Severe paracoccidioidomycosis, Autopsy. *P. brasiliensis complex.*

## Abstract

Paracoccidioidomycosis (PCM) configures a deep mycosis caused by *Paracoccidioides* spp., a neglected tropical disease. We describe a fatal case of PCM that manifested itself as osteomyelitis with thrombosis in the iliac artery in a man with frequent contact with an endemic region in Sao Paulo, Brazil. A 67-year-old man living in an endemic area presented with osteomyelitis of the femur and iliac artery thrombosis on admission to the hospital. Computed tomography of the chest showed multiple cavitated pulmonary nodules. The patient rapidly progressed to irreversible respiratory failure. The autopsy showed disseminated PCM and iliac artery thrombosis. Laboratory investigation confirmed *P. brasiliensis* infection with a strain identified in Sao Paulo as *P. brasiliensis* complex by phylogenetic analysis. Atypical PCM remains a diagnostic challenge. Increased awareness of the sites of infection and its clinical presentations will improve patient management.

## INTRODUCTION

Paracoccidioidomycosis (PCM) configures a systemic fungal infection caused by *Paracoccidioides* fungi. The disease is endemic to South and Central America, with imported cases reported in North America, Europe, Africa, and Asia by individuals who had visited or worked in endemic areas^1-3^. Brazil totals about 80% of the reported cases of PCM in South America, and estimates suggests an incidence of PCM in Brazil equal to from one to two new cases/100.000 population/year despite the lack of mandatory reporting^4^. PCM cases mostly occur in southeastern states (Sao Paulo, Rio de Janeiro, Minas Gerais, and Espirito Santo)^5^, and the average incidence of new cases per 100.000 inhabitants per year has been estimated at 0.96 (Sao Paulo city, Sao Paulo State, 1968) and 2.7 (Ribeirao Preto city, Sao Paulo State, 1980-1999)^6^.

The disease has two forms. Its acute/subacute form predominantly affects young individuals (children and adolescents) but may affect individuals aged above 30 years in association with immunosuppression (lymphadenomegaly, digestive manifestations, hepatosplenomegaly, osteoarticular involvement, and skin lesions as the main manifestations of this form of fungal infection). The chronic form occurs more often in the lungs but can affect more than one organ at a time (multifocal presentation), with the lungs, mucous membranes, and skin being the most common sites of infection^7^. Although PCM-related mortality is low, it has high morbidity, with chronic sequelae occurring in almost 50% of patients despite treatment^8^. Lack of early clinical suspicion often delays treatment, especially in cases with atypical presentations. Furthermore, a recent review of endemic mycoses showed that PCM lacks the most diagnostic tools^9^. Although PCM is endemic in Brazil, a few reports of PCM have shown vascular involvement, most of which confusing it with arteriosclerotic manifestations^10-14^.

This study describes an undiagnosed fatal case of PCM in a 67-year-old man living in an endemic area the initial manifestations of which included lower limb ischemic symptoms due to femoral osteomyelitis and extensive aortic arteritis with thrombosis. Such uncommon manifestations rarely occur in the literature and certainly contributed to delaying diagnosis^10-14^.

### Ethics

This study was approved by the Research Ethics Committee of the Clinical Hospital (HC-FMUSP) (CAPPesq Nº 3.258.615, CAAE protocol Nº 10248419.7.0000.0068). Informed consent was obtained from the patient’s family.

## CASE REPORT

The case of a 67-year old, smoker, hypertensive, dyslipidemic, male patient who lived his whole life in the urban area of Pirapora do Bom Jesus city in Sao Paulo State, Brazil, is reported in this study. The municipality is located in a valley on the banks of the Tiete River (an endemic region for PCM) with an average annual temperature of 18–22 °C, a subtropical climate and a humidity from 68 to 82% (climate-data.org). He worked in the food services industry in the urban area of the city. His hobby involved frequent freshwater fishing in this endemic region.

Since March 2019, he reported pain in his right lower leg and weight loss without a history of trauma or perforating lesions. The patient had undergone computed tomography (CT) and biopsy during hospitalization at the secondary referral hospital close to his hometown. CT showed lytic lesions in his right femur, suggesting sarcoma. However, the biopsy showed a chronic inflammatory process and tissue necrosis with unspecified fungi. He received no specific treatment. The pain in his right lower limb worsened, onsetting pain in his left lower limb, associated with non-fixed cyanosis and limb paralysis. Doppler ultrasonography showed an intra-abdominal occlusion of the inguinal vessels. On August 21, 2019, upon admission to the tertiary Hospital das Clinicas at the Faculty of Medicine of Sao Paulo, a physical examination showed cyanosis, reduced lower limb pulses, and absent femoral pulses. Angiography and magnetic resonance imaging showed partial occlusion of the infrarenal abdominal aorta into the common, internal, and external iliac arteries. The patient also had an intraosseous abscess, deep collections of the adjacent muscles, and bilateral bursitis of the psoas. On August 22, the patient underwent a chest CT scan that showed multiple nodules in both of his lungs (interpreted as septic embolism). An echocardiogram showed no cardiac vegetation as a possible focus of septic embolization of the lungs. A full anticoagulation regimen and antibiotic therapy (ceftriaxone and clindamycin) was initiated to treat osteomyelitis. Regarding the detection and identification of bacteria, culture tests for anaerobes and aerobes were carried out on the right femur lesion and peripheral blood during hospitalization with negative results. Moreover, during admission, the direct test for acid-fast bacilli (Ziehl-Nielsen) of a biopsy of the right femur lesion obtained negative results, as were the cultures for mycobacteria and fungi (biopsy of the right femur and peripheral blood). A rectal swab was also tested for carbapenem-resistant Enterobacteria, producing negative results. The bacterioscopic examination of the biopsy of the right femur showed no microorganisms.

**Figure 1:**
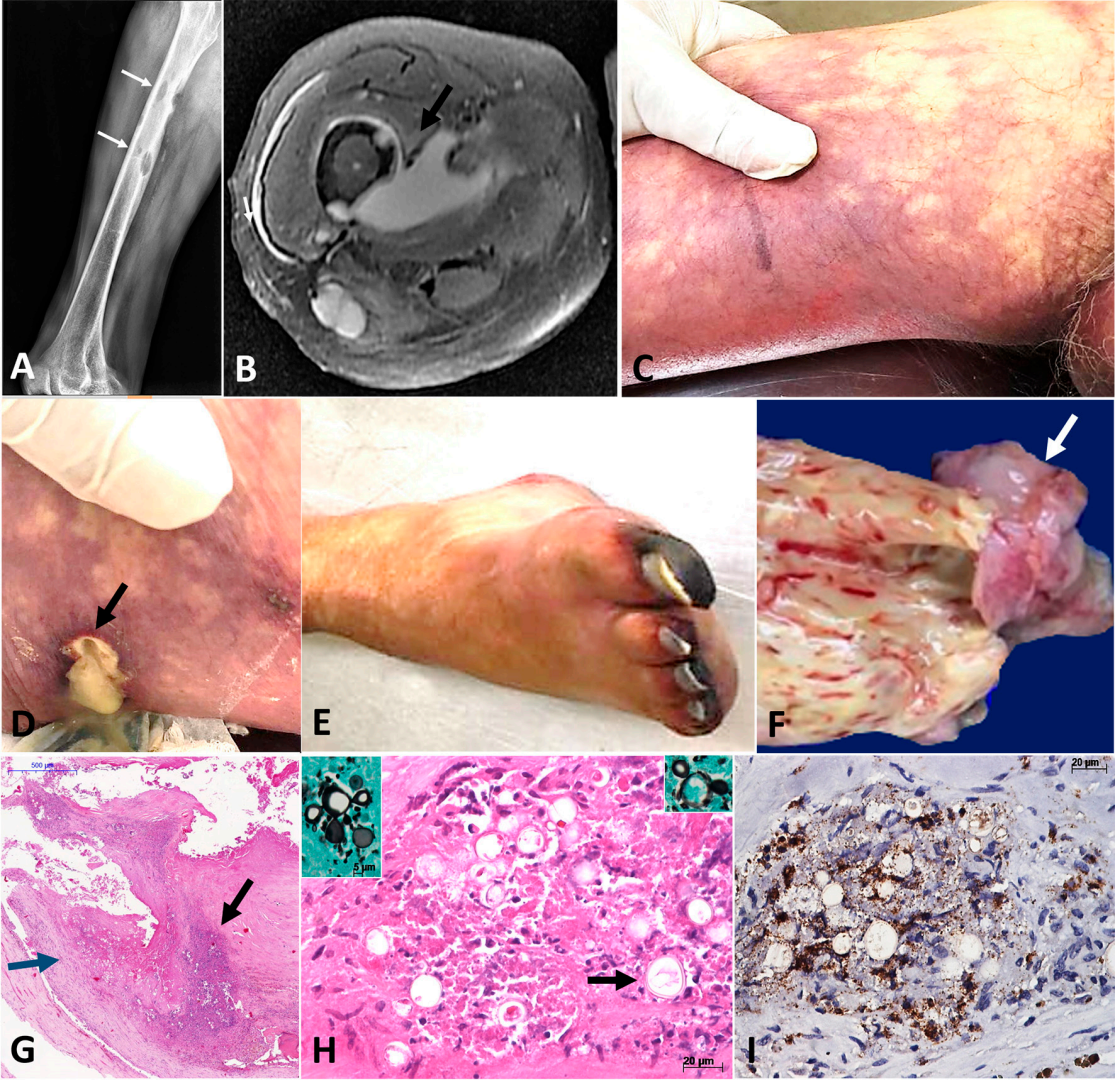
Severe paracoccidioidomycosis with arteritis and thrombosis of the iliac artery: (A) Radiography of the right femur showing multiple lytic lesions in the right femur, with periosteal reaction (arrows); (B) Nuclear magnetic resonance showing deep abscess in the right thigh associated with femoral lytic lesions; (C) Ischemia of the right thigh; (D) Fistula on the lateral area the right thigh associated with osteomyelitis, soft tissue abscess, and cutaneous infection by *Paracoccidioides*; (E) Right toe with ischemia and distal gangrene; (F) Atherosclerotic aorta with thrombus occluding right iliac artery (arrow); (G-I) Organized mycotic thrombus within the right iliac artery, with numerous yeasts in the medial layer (blue arrow) and in the thrombus (black arrow) (HE, 50×) compatible with *Paracoccidioides spp* (H: arrow, HE, 400×, and insets, by Grocott stain, 400×). The yeasts are round, with wide size range, and multiple budding (H, insets); (I) Immunohistochemistry reaction staining in brown immune cells expressing IL-17 in the aortic granuloma with *Paracoccidioides* spp. forms (Peroxydase, 400×). HE: hematoxylin-eosin.

Laboratory exams at admission showed 9.7 g/dL hemoglobin (12-15 g/dl), mild anisocytosis, 19.8 total leukocytes (5-10 × 10^3^/mm^3^), with 85% segmented cells (52-72%) and no left deviation; 990 cells/mm^3^ lymphocytes (100-3000 cells/mm^3^); and 320 C-reactive protein (<5.0 mg/L). Supplementary Table S2 shows the laboratory results at various times after presentation. The patient’s right lower limb pain evolved and he developed a fistulizing orifice that required draining the purulent material and progressively higher doses of opioids. On August 31, the patient developed acute respiratory failure and retrosternal chest pain, with symptomatic improvement after diuretic treatment and non-invasive ventilation. On September 2, he underwent an uneventful CT-guided biopsy of a lesion in his right femur (Figure 1). On the same day, he experienced significant respiratory distress, requiring intensive care unit admission. The team increased antibiotic therapy to meropenem and vancomycin in addition to amphotericin B to treat of possible fungal infection. He died on September 3.

### Autopsy findings

The autopsy showed disseminated PCM with poorly formed granulomas, histiocytes, neutrophils, and few lymphocytes without multinucleated giant cells, associated with numerous yeasts compatible with *Paracoccidiodes* spp. (Figure 1). The fungi occupied the skin along the fistulous tract from the right femur, involving the hypodermis and the entire dermis; lungs (forming cavities), aorta and right iliac artery, lymph nodes including periaortic, spleen and prostate. A 9.2-cm organized thrombus of the distal aorta extended from the infrarenal artery to the iliac bifurcation with adherence to an atherosclerotic plaque on the right iliac artery obstructing the arterial lumen, associated with ischemia of the right leg and distal necrosis of all five right toes. *Paracoccidioides* yeasts occurred in the medial layer and in the aortic/iliac thrombus (Figure 1G). Additional findings included bronchopneumonia with exudative diffuse alveolar damage (Figure 1), acute tubular necrosis, cerebral congestion, lymphoid hypoplasia in the spleen, and generalized atherosclerosis, with calcified plaques in the distal aorta and iliac arteries. A microscopic (<5 mm) pancreatic adenocarcinoma was an unexpected finding. The biopsy performed one day before death confirmed the presence of *Paracoccidioides* spp. in the femur. Moreover, our group examined the first bone biopsy (performed in the first hospital) after the autopsy, finding forms of *Paracoccidioides* spp. in the sample. We performed an immunohistochemical analysis to determine the phenotype of cells *in situ* in a granulomatous lesion of the aortic wall, using the following markers available in our laboratory: CD3, CD4, CD8, CD68, Foxp3, GATA-3, T-bet, interleukin (IL)-10, inducible nitric oxide synthase (iNOS), and IL-17. We found scarce lymphocytic response, with a few macrophages and a weak expression of iNOS, which is important for phagocytic function. Interestingly, the most expressed cytokine referred to IL-17, showing the importance of neutrophil function in the lesions in Figure 1I, Supplementary Material, Supplementary Table S1, and Supplementary Figure S1.

### Postmortem laboratory and phylogenetic investigation

**Figure 2:**
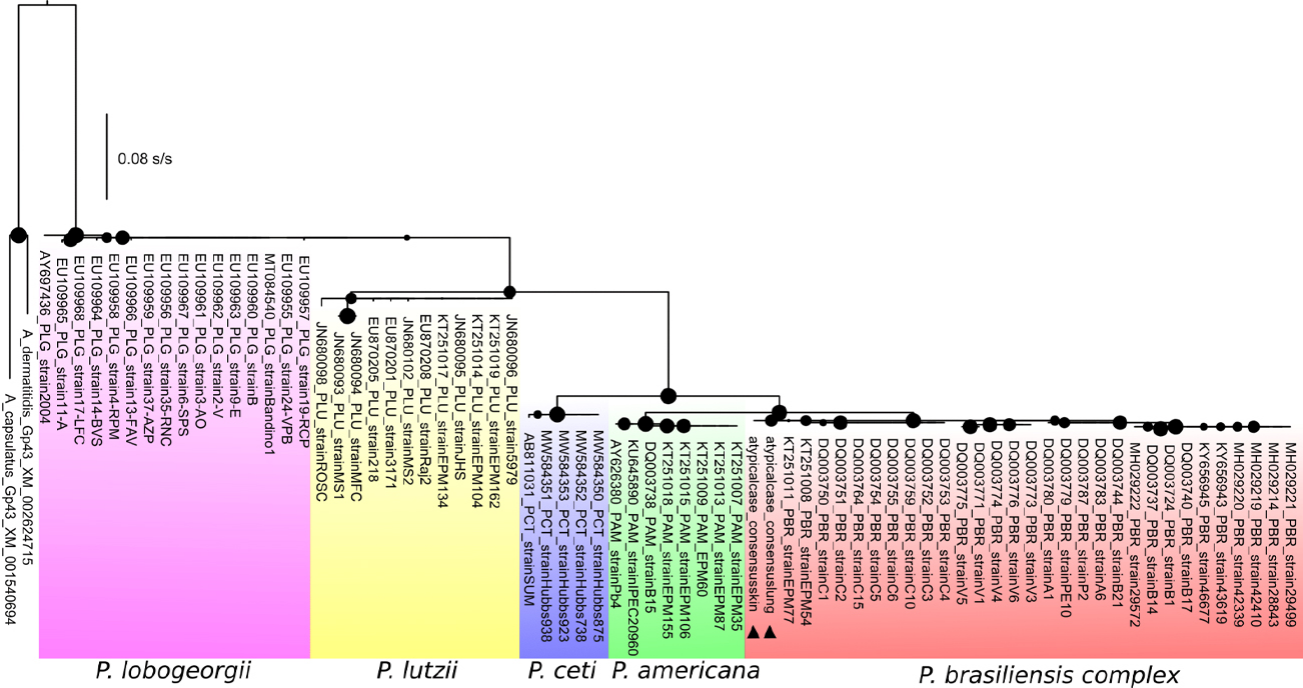
Phylogenetic analysis of *P. brasiliensis* associated with a fatal human case. The maximum likelihood phylogenetic tree of the genus *Paracoccidioides* was based on partial *gp43* gene sequences (n = 75). The black circles represent the bootstrap support values, and their sizes vary according to the support value (0 to 100). The black triangles highlight the samples characterized here from the atypical case of PCM, in which the two samples cluster with sequences characterized a *P. brasiliensis* complex.

*Postmortem* serologic and sequencing analysis identified *P. brasiliensis*. This research deposited the sequences on GenBank under accession numbers MT346581(skin) and MT346580 (lung). Phylogenetic inference using partial gp43 sequences showed that the *P. brasiliensis* complex grouped the two sequences (Figure 2). Sequencing and mycological analysis of samples from lung and skin tissues showed co-infection with other fungal pathogens *Trichosporon montividense* (MT322616) and *Candida albicans* (MT322617) (Supplementary Figure S2).

## DISCUSSION

This case reports concerns an older male adult with rare manifestations of chronic PCM, presenting lung lesions, femoral osteomyelitis, abdominal lymphadenitis, and aortitis, leading to iliac artery thrombosis. The initial presentation of the infection included lower extremity symptoms, which contributed to delaying diagnosis. Rare descriptions of PCM have involved it in the osteoarticular system and large vessels such as the aorta and iliac arteries in the same patient. Our case of *Paracoccidioides* iliac thrombosis confirms the observations of Brass in 1969, who described aortitis and granulomatous iliac arteritis associated with infection by the fungus in three cases of disseminated disease involving the lungs, adrenals, lymph nodes (including the periaortic nodes). Noteworthy that it is often superimposed on atheromatous plaques. The presence of fungi in the patient’s thrombus and arterial wall suggests that the granulomatous inflammatory process contributes to endothelial damage and thrombosis, possibly favored by the lipid environment of atherosclerotic lesions, as proposed by Brass^11^. The aortic/iliac infection in our case may be multifactorial: hematogenous dissemination of fungi; infection of an arterial wall with severe atherosclerotic disease; contiguity from an infected right femur or periaortic lymph nodes; and immune dysfunction, as we observed in the morphology of the granulomas and in *in situ* immune phenotyping. Interestingly, the autopsy showed pancreatic adenocarcinoma, which may have predisposed the patient to thrombotic phenomena. In this case, the autopsy was crucial for the final diagnosis.

Although PCM disease is endemic in Brazil, the literature has no recent descriptions of vascular involvement as in our case. The literature has described less than 10 cases, the last of which dating to 1998^10-14^. All reports included adult patients, whose diagnosis was delayed or made only at autopsy. PCM aortitis produces confusion with arteriosclerotic manifestations, leading to a lack of early clinical suspicion and delayed or unspecific treatment.

Osteoarticular PCM also remains rarely described. The few studies addressing it report common bone infections in children or younger adults in the acute/subacute phase of the disease^15,16^. Most described cases occurred in Brazil. A hypothesis in Sevarese *et al*.^16^ states that skin lesions and pulmonary involvement usually dominate the presentation when PCM is diagnosed without an active and systematic search for bone lesions.

In this patient, the autopsy found a microscopic adenocarcinoma of the pancreas. Concomitant PCM and solid tumors have occurred in from 0.16 to 11% of PCM cases^17^. Most tumors are respiratory or gastrointestinal cancers, sometimes at the same site of infection. It remains unknown whether predisposing factors such as smoking and alcoholism are common to both conditions or whether PCM may be an additive factor for cancer development due to the chronic antigenic stimulation of epithelial cells^7,17^. Unfortunately, given the lack of suspicion of paracoccidioidomycosis and the patient’s fulminant presentation at our hospital, no further investigation of coagulopathy or immune function was performed. The aortic/iliac thrombosis in the right leg was clinically attributed to complicated atherosclerosis. We are unable to definitively rule out the possibility that the pancreatic microcarcinoma may have constituted a factor for thrombotic coagulopathy and immune cell dysfunction in this case. In other cases, PCM may mimic a solid malignancy, further delaying diagnoses^18,19^.

Although classical laboratory techniques provide valuable information by visualizing the fungal pathogen *Paracoccidioides* spp, they have no significant discriminatory power on the type of species. Several PCR-based methods can detect polymorphisms in the DNA of *Paracoccidioides* and support species identification, mainly sequencing and taxonomic analysis, enabling the location of the *P. brasiliensis* complex strain as originating from the endemic region of Sao Paulo^20^. The results of phylogenetic reconstruction using gp43 sequences confirmed the diagnosis of the etiologic agent, and grouping the two sequences from different tissues confirmed that they belonged to the same strain. The long latency before the clinical picture of PCM infection emerges complicates efforts to trace clinical symptoms to events associated with initial exposure. Fungal sexual reproduction and competitive selection increase in this environment, with a greater likelihood of the emergence of more virulent variants^2,5,20^. These sequences may be related to PCM survival strategies in saprophytic or specific host immunities, justifying its atypical clinical picture. Moreover, PCM sequences contribute to updating maps of endemic fungal regions, which may improve global PCM surveillance, according to the World Health Organization recognition of *Paracoccidioides* spp. as a priority fungal pathogen^1^.

A limitation of our study is that *Paracoccidioides* spp. was not isolated from the patient’s cultured specimens. However, histological analysis identified the fungus, sequencing it using fresh frozen samples and formalin-fixed paraffin-embedded tissue from various tissues. Cultures also detected *T. montividense* and *C. albicans* (confirmed by sequencing). These faster growing pathogens may have inhibited the growth of *P. brasiliensis* in culture by competing for nutrients, explaining our negative results.

The results of our study should raise the clinical suspicion of PCM and lead to more rational and precise anti-infective treatments, especially for patients who are difficult to diagnose by conventional methods. This will positively impact risk.

## CONCLUSION

This report describes a rare form of chronic PCM with osteoarticular involvement of the femur, aortitis, and iliac artery thrombosis in an older male adult with frequent contact with an endemic region of São Paulo, Brazil. Increased awareness of the transmission sites and different clinical presentations of this neglected tropical disease may improve patient management.
